# Molecular and cellular correlates in Kv channel clustering: entropy-based regulation of cluster ion channel density

**DOI:** 10.1038/s41598-020-68003-4

**Published:** 2020-07-09

**Authors:** Limor Lewin, Esraa Nsasra, Ella Golbary, Uzi Hadad, Irit Orr, Ofer Yifrach

**Affiliations:** 1 Department of Life Sciences and the Zlotowski Center for Neurosciences, Ben-Gurion University of the Negev, P.O.B. 653, 84105 Beer Sheva, Israel; 2 Ilse Katz Institute for Nanoscale Science & Technology, Ben-Gurion University of the Negev, P.O.B. 653, 84105 Beer Sheva, Israel

**Keywords:** Potassium channels, Intrinsically disordered proteins

## Abstract

Scaffold protein-mediated ion channel clustering at unique membrane sites is important for electrical signaling. Yet, the mechanism(s) by which scaffold protein-ion channel interactions lead to channel clustering or how cluster ion channel density is regulated is mostly not known. The voltage-activated potassium channel (Kv) represents an excellent model to address these questions as the mechanism underlying its interaction with the post-synaptic density 95 (PSD-95) scaffold protein is known to be controlled by the length of the extended ‘ball and chain’ sequence comprising the C-terminal channel region. Here, using sub-diffraction high-resolution imaging microscopy, we show that Kv channel ‘chain’ length regulates Kv channel density with a ‘bell’-shaped dependence, reflecting a balance between thermodynamic considerations controlling ‘chain’ recruitment by PSD-95 and steric hindrance due to the spatial proximity of multiple channel molecules. Our results thus reveal an entropy-based mode of channel cluster density regulation that mirrors the entropy-based regulation of the Kv channel-PSD-95 interaction. The implications of these findings for electrical signaling are discussed.

## Introduction

Action potential generation, propagation and the evoked synaptic potential all rely on precisely timed events associated with activation and inactivation gating transitions of voltage-dependent Na^+^ and K^+^ channels, clustered in multiple copies at unique membrane sites, such as the initial segment of an axon, nodes of Ranvier, pre-synaptic terminals or at the post-synaptic density (PSD)^1–3^. Changes in either ionic current shape or density, reflecting changes in temporal and spatial dimensions, respectively, affect action potential shape and frequency and may lead a neuron to change its mode of firing^4–9^. Despite emerging evidence attesting to the importance of ion channel density for efficient electrical signaling^7^ and information encoding^7–9^, little is currently known of the clustering process itself or its regulation. It is, however, clear that ion channel clustering is an active process, involving the interaction of a channel with a specific member of one of several scaffold protein families. For example, Nav channel clustering at nodes of Ranvier is mediated by interaction of the channel with the ankyrin G scaffold protein^10,11^. Kv channel clustering at the PSD of excitatory synapses (of Drosophila melanogaster but not of mammalians), on the other hand, is mediated by binding to the PSD-95 synapse-associated scaffold protein^12–15^. Yet, the mechanism by which these elementary binding events lead to the clustering of ion channel molecules in a restricted area of the membrane remains unclear. Furthermore, it is also generally not known if and how ion channel membrane density is regulated in the spatial and/or temporal dimensions. In the absence of a molecular mechanism describing the channel protein-scaffold protein interaction, bridging this molecular-cellular gap to understand ion channel clustering has proven challenging.

**Figure 1:**
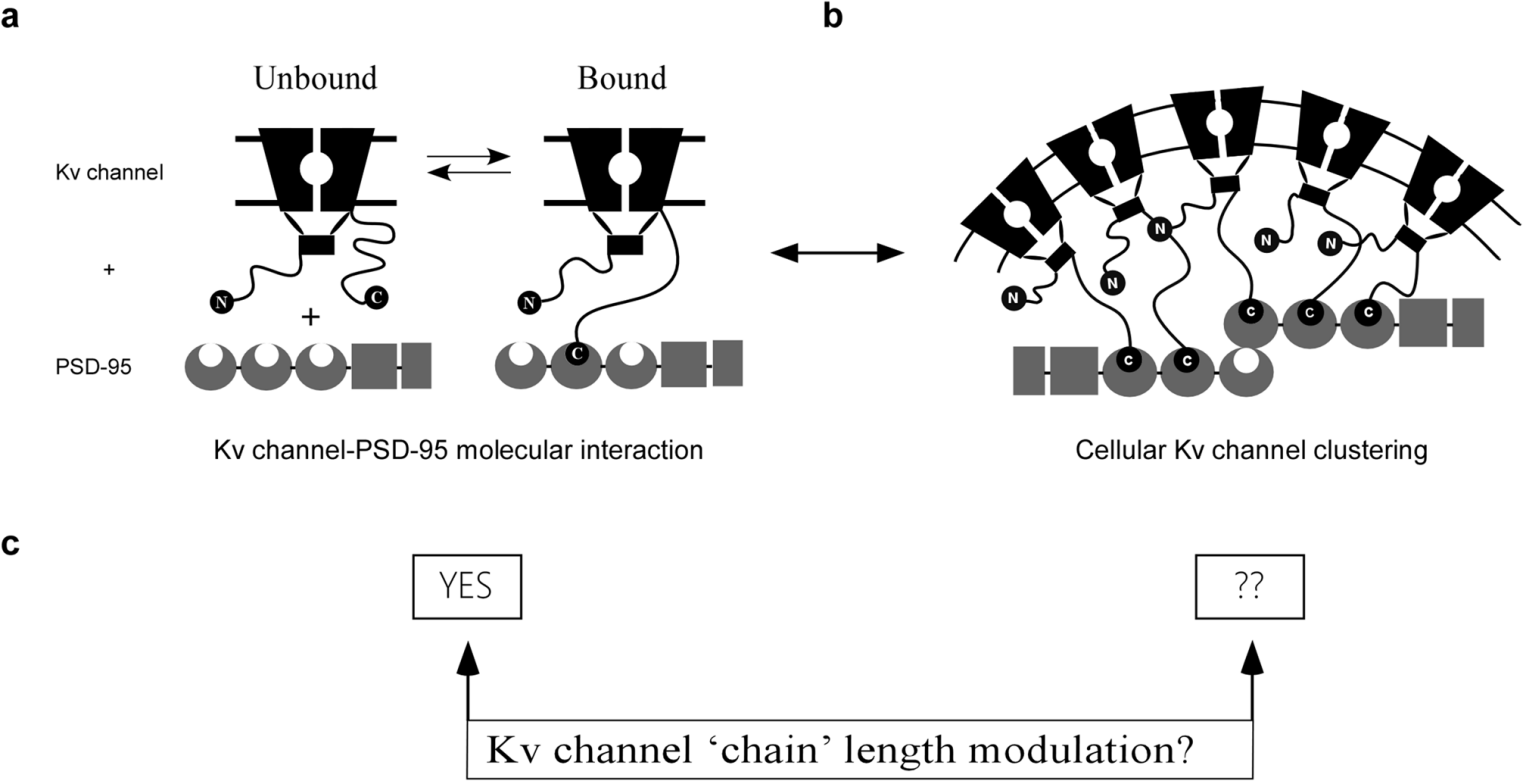
A ‘ball and chain’ mechanism for Kv channel clustering? (**a**) Schematic representation of the ‘ball and chain’ mechanism for Kv channel binding to the PSD-95 scaffold protein (for clarity, only two channel subunits are presented). In this mechanism, the intrinsically disordered 'chain' at the Kv channel C-terminal binds PSD-95 upon interaction of the ‘ball’ PDZ binding motif to PSD-95 PDZ domain(s). This mechanism is reminiscent of the mechanism underlying fast channel inactivation. In this former mechanism, the length of the Kv channel C terminal 'chain' governs its interaction with PSD-95 in a manner which is entropy-controlled. Given the ability of PSD-95 to aggregate and the stoichiometry of the interaction, channel clustering at unique site results (**b**). Whether or not Kv channel 'chain' length regulates channel density within clustering sites was addressed in the current study (**c**). The rectangular shape corresponds to the assembly T1 domain of the channel while the membrane-embedded portion corresponds to the channel voltage-sensor and pore domains. The crescent, box and rectangular shapes represent the PDZ, SH3 and guanylate kinase-like domains of the PSD-95 protein, respectively. Figure panels (**a**,**b**) were adapted from ref.^27^ with permission.

The prototypical *Shaker* Kv channel protein is an excellent model system to address these questions since the molecular mechanism underlying its interaction with the PSD-95 scaffold protein is known^16,17^. According to the ‘ball and chain’ mechanism that describes this interaction, the random walk motion of the unstructured C-terminal channel ‘chain’, bearing a conserved six amino acid PDZ-binding motif (the ‘ball’) at its tip, recruits the PSD-95 scaffold protein partner (Fig. 1a)^16,17^ in a manner analogous to the role of the N-terminal tail in regulating channel fast inactivation^18^. Evidence supporting this mechanism primarily relies on the ‘chain’-length dependence of thermodynamic and kinetic parameters controlling the Kv channel-PSD-95 interaction^17,19,20^, in a manner predicted by polymer chain theory^21^. For example, both the affinity (*K*_eq_) and entropy (∆*S*) of the binding reaction were found to linearly depend on Kv channel ‘chain’ length^17^. Furthermore, the rate constant of association between both proteins revealed an expected power law dependence on 'chain' length^17,19,20^. Based on these and other findings, it has been argued that the Kv channel C-terminal sequence functions as an entropic clock^22,23^ that times PSD-95 binding, with ‘chain’ length acting as the hands of the clock^17,20^. Indeed, alternative splicing of the *Shaker* channel gene only occurs at either the N- or C-terminal ‘chains’ to produce natural channel variants presenting different ‘chain’ lengths^24,25^. These variant 'chains' give rise to distinct binding kinetics in inactivation^18,26^ or PSD-95 binding^17,20^, respectively.

The ‘ball and chain’ mechanism that describes the Kv channel-PSD-95 interaction is molecular in essence, and as such, reveals no information on Kv channel clustering. One can thus ask what is the cellular correlate(s) of the ‘ball and chain’ mechanism for channel-scaffold protein binding, if any (Fig. 1b)? Previous studies indicated that Kv channel 'chain' length determines the level of channel expression within clusters^20^, however, the question of whether or not Kv channel ‘chain’ length regulates Kv channel density remained unaddressed (Fig. 1c). To answer this question, we used sub-diffraction high-resolution confocal imaging microscopy of PSD-95-mediated Kv channel clustering, combined with quantitative clustering analysis, to calculate cluster ion channel densities (i.e. the density of ion channel molecules within a cluster). Our study revealed that Kv channel ‘chain’ length regulates cluster Kv channel membrane density with a ‘bell’-shaped dependence, reflecting the balance between steric hindrance and thermodynamic considerations controlling ‘chain’ recruitment by the PSD-95 scaffold protein. Our results thus provide an example of how a particular molecular mechanism describing a protein–protein interaction is manifested at the cellular level to modulate membrane ion channel density. Such modulation has important implications for electrical signaling in the nervous system.

## Results

### High-resolution confocal imaging microscopy of *Shaker* Kv channel clustering

Confocal imaging microscopy was previously used to describe Kv channel clustering in several heterologous expression systems^12,14,16^. Such studies focused on identifying the binding determinants of the Kv channel and PSD-95 variants that are important for channel cell surface expression and clustering. These efforts, however, did not provide quantitative insight into Kv channel clustering, primarily due to the low resolution of the imaging techniques employed and the absence of a molecular mechanism describing the channel-scaffold protein interaction. In the present study, we used Airyscan, a sub-diffraction high-resolution laser-scanning confocal microscope^28–30^, to examine PSD-95-mediated *Shaker* Kv channel clustering in a model neuronal cell expression system (SH-SY5Y neuroblastoma cells). This experimental setup offers two main advantages. First, the SH-SY5Y cells readily express both the Kv channel and PSD-95 proteins and spread well on a cover slip, allowing for imaging of a major portion of the cell surface. Second, the Airyscan detector used in current study provides improved lateral resolution (~ 150 nm) and signal to noise ratio, as compared to conventional confocal microscopes. Under these conditions, accurate and straightforward analysis of channel clustering was allowed. In particular, it was possible to quantitatively evaluate Kv channel membrane cluster area size and the integrated fluorescence signal within the cluster, thus allowing us to calculate the averaged channel-associated signal intensity, an important parameter that is directly related to the average density of Kv channel molecules per cluster membrane area^31^. Given these advantages and the fact that the *Shaker* Kv channel-PSD-95 interaction can be accurately described by a ‘ball and chain’ molecular mechanism, it was possible to address whether Kv channel entropic ‘chain’ length indeed regulates membrane Kv channel density within clusters.

**Figure 2:**
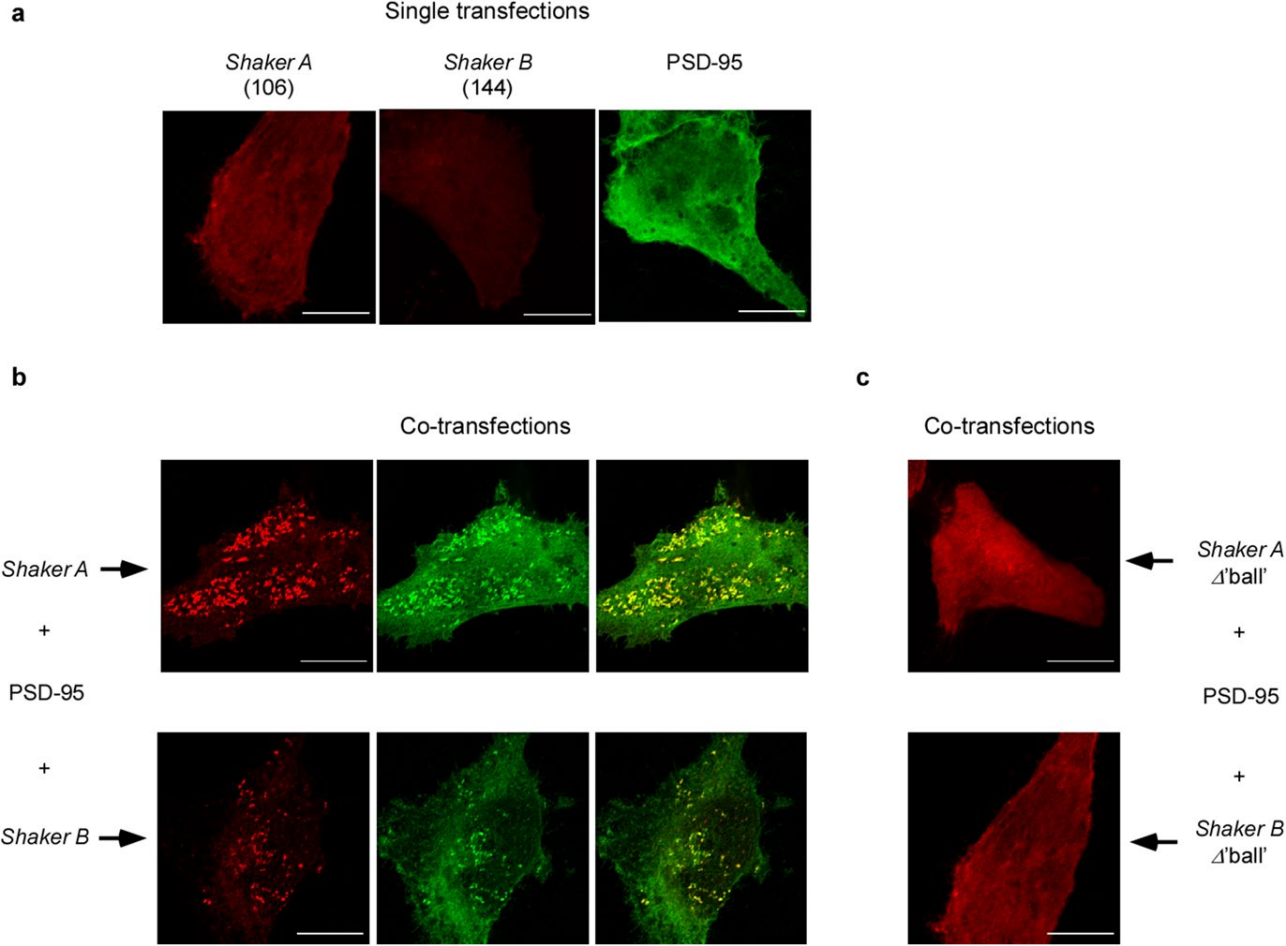
Cell expression and clustering patterns of the native* A* and *B Shaker* Kv channel variants. (**a**) High-resolution confocal microscopy images of SH-SY5Y neuroblastoma cells expressing either the native *Shaker A* or *B* Kv channel variants alone or the PSD-95-GFP scaffold protein partner. Typical images of cells co-expressing either the *A* or *B* native channel variants and PSD-95-GFP are shown in the upper and lower sections of (**b**). Three images are shown for each cell, with the red channel-associated fluorescent signal shown in the left column, the green PSD-95-associated signal presented in the middle columns, and the merged shown in the right column. (**c**) Typical images of SH-SY5Y cells co-expressing PSD-95-GFP and either the Shaker *A* or *B* native Kv channel variants lacking the terminal 'ball' PDZ binding motif (∆'ball'). Scale bars in panels (**a**) to (**c**) correspond to 10 μm. Numbers next to each channel notation indicate C-terminal amino acid ‘chain’ length.

We began by analyzing the clustering phenotypes of the two short and long C-terminal native ‘chain’ variants of the *Shaker* Kv channel (the *A* and *B* splice variants, respectively comprising 106 and 144 amino acids and both carrying an identical terminal PDZ-binding ‘ball’ motif). These variants exhibited distinct affinities to PSD-95, with the *A* variant presenting higher equilibrium and rate constants for association to PSD-95^17^. Neuroblastoma cells were transfected to express FLAG-tagged short or long native Kv channel ‘chain’ variants, either alone or together with PSD-95-GFP. Following cell fixation, permeabilization and immunostaining of cytoplasmic channel epitopes, the cells were imaged by focusing directly on the ‘cover slip’-attached basal membrane plane. Under such an experimental paradigm, one cannot unambiguously discern whether the *Shaker* Kv channels are expressed on the cell membrane surface or in a near-membrane intra-cellular vesicle pool. Whether or not one considers the near-membrane or surface expression does not, however, change the mechanistic insight gained in this work with respect to channel clustering. Typical confocal microscopy images of the *A* and *B* channel variants are provided in Fig. 2. Control experiments involving cells transfected to express either of the *Shaker A* or *B* channels alone or just PSD-95-GFP showed homogeneous and diffuse labeling in the vicinity of the plasma membrane, reflected by the* Shaker* channel-associated red fluorescent pattern or the palmitoylated PSD-95-associated green fluorescent signal^32^ (Fig. 2a). However, co-expression of PSD-95-GFP and either of the *Shaker* channel variants (Fig. 2b) resulted in a speckled pattern. As judged by the membrane-associated yellow coloring of the merged images, this pattern reflects protein co-localization and clustering. Indeed, the clustering pattern detected is very similar to that observed when the Kv channel-PSD-95 pair was co-expressed in other heterologous expression systems^12,14^. Furthermore, the speckled co-localization pattern was absent in control experiments where PSD-95 was co-transfected with *A* and *B* channel mutants lacking the terminal SIETDV clustering ‘ball' (i.e., the PDZ-binding motif), a region absolutely required for PSD-95 binding (Fig. 2c). In this case, the images of either protein exhibited a diffuse pattern at or near the cell membrane, as also observed in experiments where only single wild type channel variants were expressed (Fig. 2a). Relatively similar patterns of PSD-95-mediated *A* or *B* channel expression were also observed when the protein-expressing SH-SY5Y cells were imaged in a total internal reflection (TIRF) mode using a super-resolution microscope (see Methods). As can be seen in Supplementary Fig. S1, the red Kv channel-associated signal appeared to be completely within the 100 nm TIRF zone of the microscope, suggesting that the immediately adjacent membrane vesicle pool or the membrane surface itself is being imaged.

**Figure 3:**
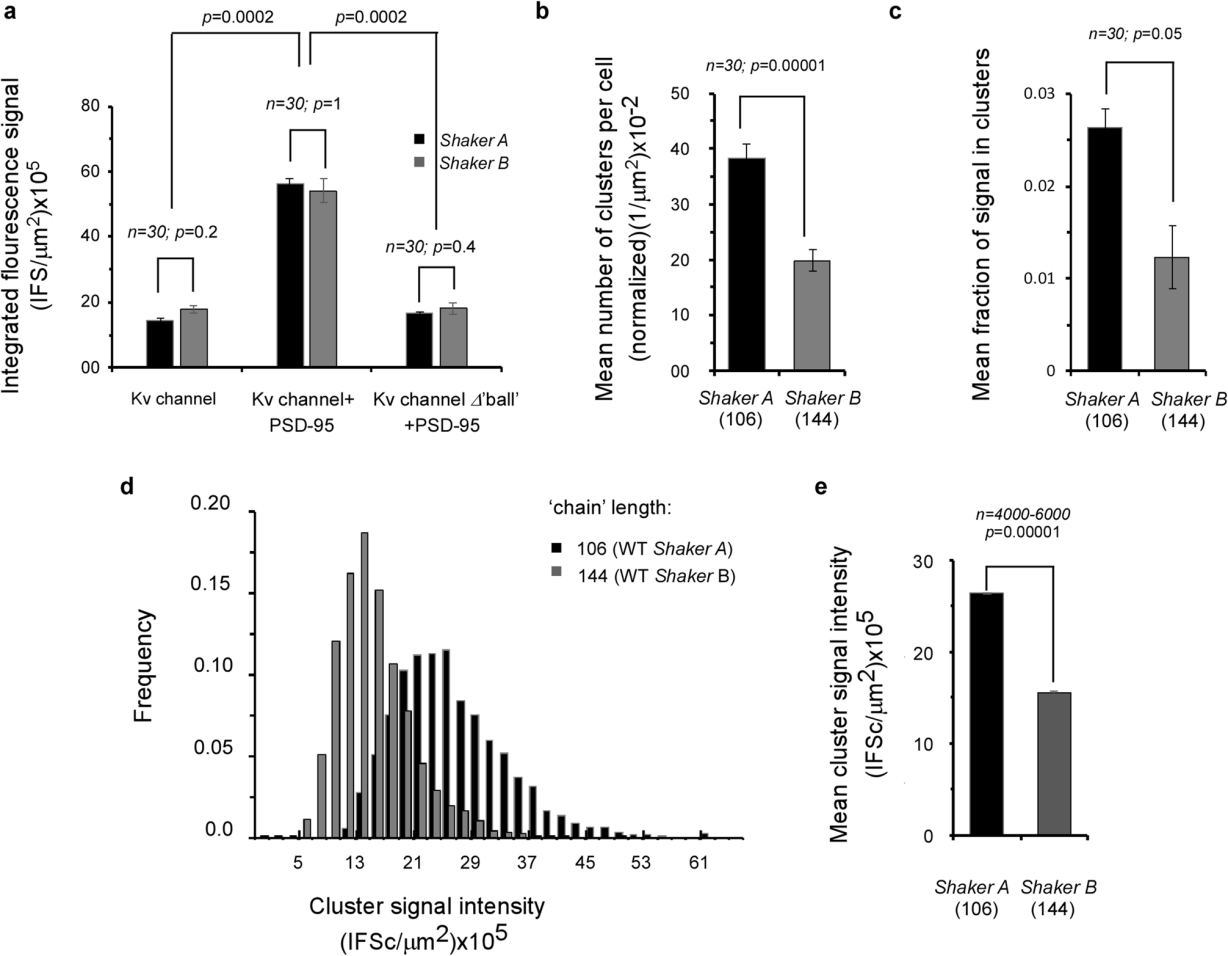
The native ‘chain’ length channel variants exhibit differences in clustering attributes. (**a**) Comparison of mean expression levels (reflected in the integrated cell fluorescence signal and normalized to cell area) of the *A* and *B* Kv channel variants in the absence (left) or presence (middle) of PSD-95. Expression levels of *A* and *B* mutants lacking the 'ball’ motif, along with PSD-95, are shown in the right panel. (**b**) Comparison of the number of clusters per cell (normalized to cell area) of the *A* and *B* Kv channel variants. (**c**) Comparison of the mean fraction of *A* and *B* Kv channel variants expressed in clustering sites. (**d**) Cluster Kv channel density distributions of the *A* and *B* variants (evaluated by cluster signal intensity), as supported by PSD-95. Distributions are in steps of 2 (× 10^5^) IFSc/μm^2^ are presented (*n* = 4,000–6,000 clusters). (**e**) Comparison of the mean value of PSD-95-mediated *A* or *B* cluster signal intensity.

To quantify the effect(s) of the native *A* and *B* ‘chains’ on PSD-95-mediated Kv expression and clustering seen on Fig. 2, we employed the clustering metrics methodology described in the Methods to evaluate the following four characteristics: (1) the total cell fluorescence signal (normalized to cell area), related to the overall channel expression level; (2) the fraction of channel-associated fluorescence signal observed in clustering sites; (3) the number of clusters per cell (normalized to cell area); and (4) cluster fluorescence signal intensity, related to ion channel density within clusters. The results of such analysis are presented in Fig. 3. As can be seen in Fig. 3a, the two native channel variants exhibited similar expression levels under all experimental conditions considered. Not surprisingly, for both variants, higher expression was observed when the channel was co-expressed with PSD-95 (*n* = 30; *p* = 0.0002)^14^; this level was reduced to basal values when channel variants lacking the terminal ‘ball’ motif were used (*n* = 30; *p* = 0.0002). In contrast, the two ‘chain’-length channel variants exhibited differences in the mean number of clusters per cell and the fraction of channels in clusters, with the short ‘chain’-bearing high affinity *A* variant presenting higher values than the long chain-presenting low affinity *B* variant (Fig. 3b,c, respectively). Last, and most importantly, statistical analysis of 4,000–6,000 clusters of each variant obtained from 30 individual cells revealed that the distribution of cluster ion channel densities of the two variants (as reflected by fluorescence signal intensities) spanned different ranges, with that of the short ‘chain’ *A* variant being displaced towards higher channel densities (Fig. 3d). This is further reflected in the mean value for cluster signal intensity being almost twice as large for the *A* variant than for the longer ‘chain’ *B* variant (Fig. 3e) (*n* =  5,000; *p* = 0.00001).

### Kv channel C-terminal ‘chain’ length affects cluster ion channel density

**Figure 4:**
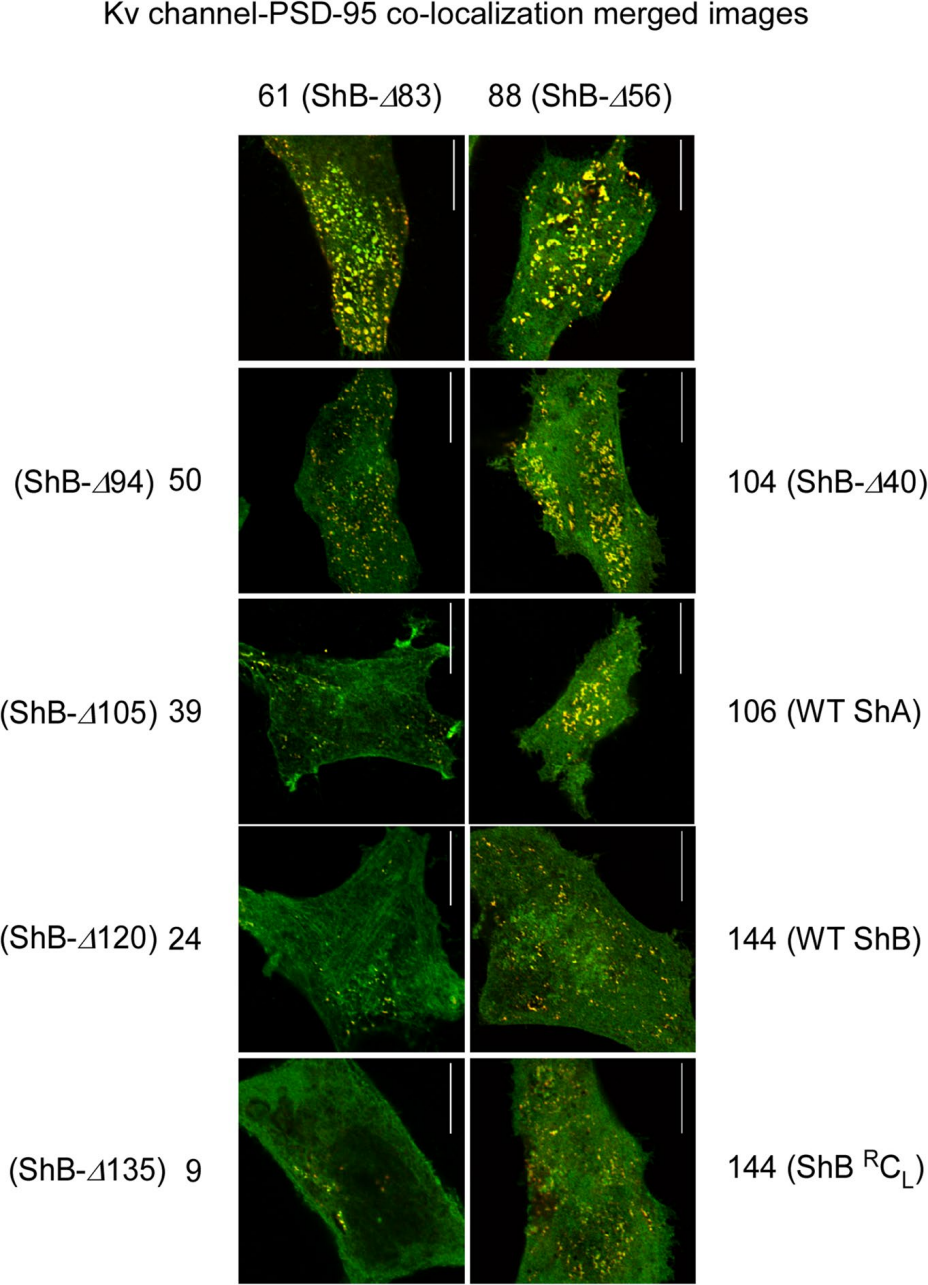
Cell expression and clustering of all *Shaker* Kv channel 'chain' length variants. Typical high-resolution confocal microscopy images of cells co-expressing PSD-95-GFP and either native or modified Kv channel 'chain'-length variants presenting a length series of 9, 24, 39, 50, 61, 88, 104, 106 and 144 amino acids. For each cell, only the merged yellow co-localization image is shown (See Supplementary Fig. S2 for all typical red (channel-associated), green (PSD-95-associated) and yellow signal (merged) images). Scale bars correspond to 10 μm. Numbers next to each channel notation indicate C-terminal 'chain' length.

**Figure 5:**
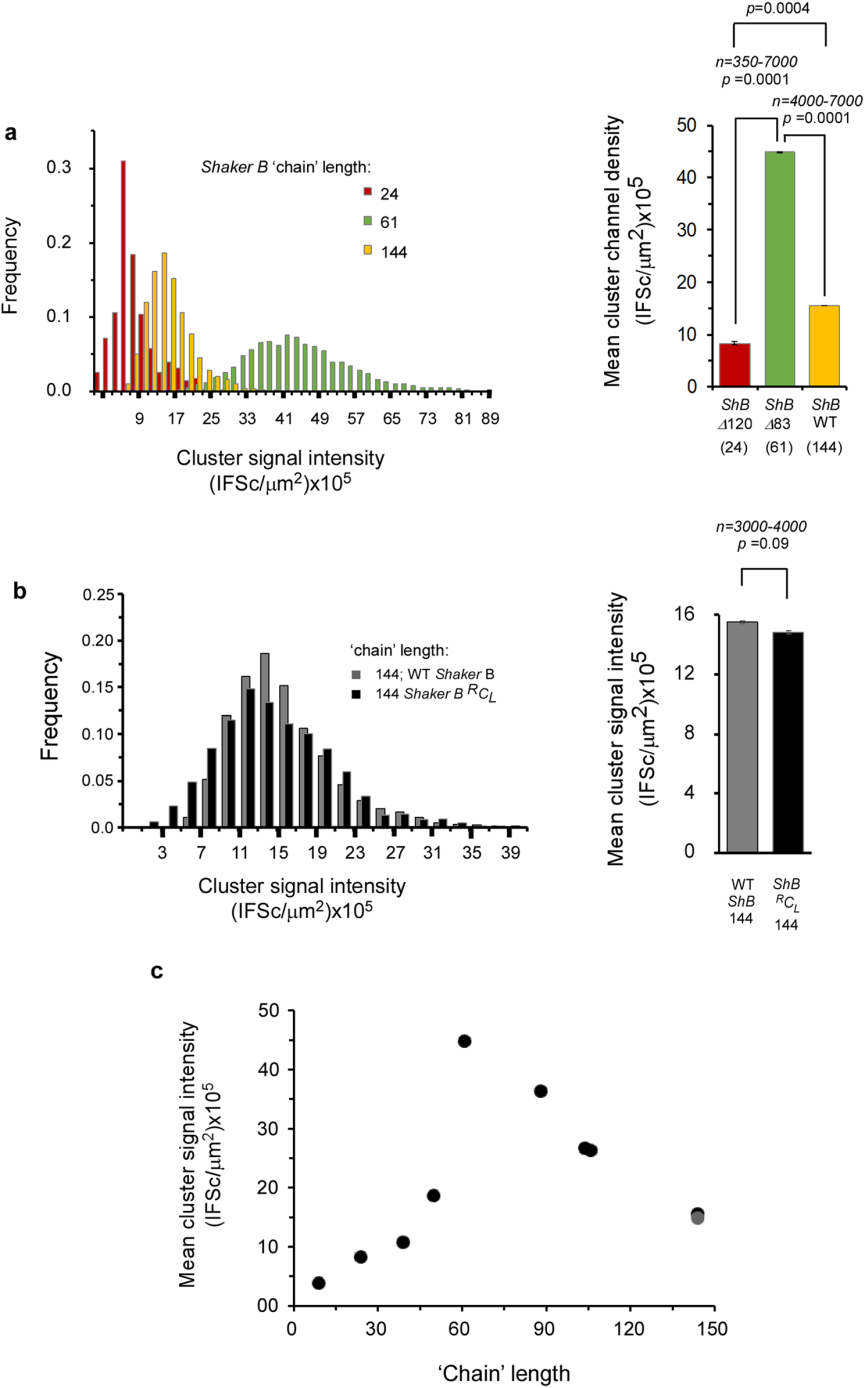
‘Chain’-length modulation of cluster Kv channel density. (**a**) Cluster Kv channel density distribution for variants presenting chain lengths of 24, 61 and 144 amino acids, as evaluated by cluster signal intensity and supported by PSD-95. Distributions in steps of 2 (× 10^5^) IFSc/μm^2^ are presented (*n* = 350–7,000 clusters). The mean values of cluster ion channel density for the different variants are compared in the panel to the right of the distributions panel. All differences were found to be statistically significant, based on ANOVA analysis (the *p*-values for all three comparisons are smaller than 0.00001). (**b**) Comparison of cluster Kv channel density distribution of the wild type or reversed C terminal ‘chain’ *Shaker B* channels exhibiting identical (144 amino acid long) C-terminal chain lengths and composition (*n* = 3,000–4,000). The mean values of cluster ion channel density for the two variants are compared in the panel to the right. No significant difference was observed between the two variants (*p*-value = 0.09). (**c**) Dependence of the mean value of PSD-95-mediated cluster Kv channel density on C-terminal ‘chain’ length. Differences in cluster ion channel density were all found to be statistically significant (other than for channel chains of similar lengths), based on an ANOVA test (*n* = 350–7,500; *p* < 0.00002). The gray data point indicates the *Shaker B* reversed C terminal ‘chain’.

The results above imply that Kv channel C-terminal ‘chain’ length modulates cluster Kv channel density. To test this assertion, we used a series of Kv channel variants presenting different C-terminal ‘chain’ lengths, ranging from 9 to 144 amino acids, all presenting the same terminal PDZ-binding ‘ball’ motif, and examined their effects on PSD-95-mediated clustering metrics under identical conditions. These ‘chain’ length variants were generated by artificially shortening the C-terminal ‘chain’ of the *B* variant by 40, 83, 120 or 135 amino acids^17^, or by replacing the original native clustering (C) ‘chains’ with short (S) or long (L) native versions of the N-terminal inactivation (I) ‘chain’, known to be entropic random ‘chains’ and previously shown as being able to replace the original native C-terminal chains with respect to PSD-95 binding and PSD-95-mediated channel expression and clustering^20^. Together with the native *A* and *B* channel variants, the channel series thus presented C-terminal ‘chain’ lengths of 9, 24, 39, 50, 61, 88, 104, 106 and 144 amino acids. The series further included a *B* channel ‘chain’ variant presenting a sequence with reversed amino acid order at the C-terminus (^R^C_L_). The *B* channel C_L_ and ^R^C_L_ 'chain' variants exhibited identical ‘chain’ lengths and amino acid composition, thus serving as internal clustering controls. Typical high-resolution confocal microscopy images of neuroblastoma cells transfected to express each of the ‘chain’ length variants, together with PSD-95-GFP, are presented in Supplementary Fig. S2, with the corresponding merged co-localization yellow images of all variants compared in Fig. 4. As can be seen, all of the different ‘chain’ length variants exhibited a speckled co-localization pattern when expressed with the PSD-95 protein, although distinct co-localization and clustering patterns were observed in each case (for the set of three images for each variant, see Supplementary Fig. S2). Multiple images (*n* = 30) of each channel variant were subjected to the clustering metrics methodology described above to determine whether or not systematic differences in cluster ion channel densities were observed as a function of ‘chain’ length. The resulting distributions of cluster ion channel density (reflected as signal intensity) of all variants are presented in Supplementary Fig. S3 and are further compared in Fig. 5. The results reveal that the distributions of cluster ion channel density of all ‘chain’-length variants appeared normal and presented different cluster density ranges (Supplementary Fig. S3). This can be clearly seen in Fig. 5a (left panel), where the distributions of cluster ion channel density of three Kv channel ‘chain’ variants presenting ‘chain’ lengths of 24, 61 or 144 amino acids are directly compared. This is further reflected by the mean cluster ion channel densities calculated for these variants (Fig. 5a, right panel). Furthermore, Kv channel variants presenting identical or very similar ‘chain’ lengths exhibited similar distributions of cluster ion channel density. For example, the *Shaker B* (C_L_) or the *Shaker* B reversed (^R^C_L_) 144 amino acid-long ‘chain’ channels, presenting identical lengths and composition, exhibited very similar cluster ion channel density distributions (Fig. 5b, left panel), as further reflected in the similar mean value of cluster ion channel cluster densities calculated for these variants (Fig. 5b, right panel). The same was true for the wild type *Shaker A* and mutant *Shaker B* ∆*40* deletion channels presenting 106 and 104 amino acid-long ‘chains’, respectively (Supplementary Fig. S4). Here again, a very similar mean value of cluster channel density was obtained (right panel). The mean values of cluster ion channel density for all different channel variants used in the current study were calculated and all were found to differ (*n* = 350–7,000; *p* < 0.0001 in an ANOVA test; see Methods), except for the case of control channel pairs exhibiting identical or almost identical ‘chain’ lengths (*p* = 0.09 or higher).

Plotting the mean value of cluster ion channel density for all Kv channel variants against C-terminal ‘chain’ length revealed an interesting bell-shaped-like dependence with an approximate maximum point around a ‘chain’ length of 61 amino acids (Fig. 5c). Starting from the 144 residue-long 'chain' of the wild type *Shaker B* channel (or its C-terminal reversed version (gray data point)), systematic shortening of the C-terminal ‘chain’ to the 61 residue optimum point resulted in a linear increase in the mean cluster channel density (Fig. 5c). Further shortening of the ‘chain’ to a length of 50 residues or less led to a significant decrease in the mean values of cluster ion channel densities. This rise and fall in cluster ion channel density around the optimum point is nicely illustrated when inspecting the distributions of cluster ion channel densities of the channel variants presenting 'chain' lengths of 24, 61 and 144 amino acids (Fig. 5a). A similar bell-shaped dependence was also observed for the mean number of clusters per cell and the fraction of channels in clusters but not for the normalized mean expression level parameter (Supplementary Fig. S5), arguing that Kv channel chain length affects PSD-95 clustering metrics, yet not cell expression levels. The values of all clustering attributes for the Kv channel 'chain' length variants are reported in Table 1.

**Table 1:** Analysis of PSD-95-mediated Kv channel clustering.

Kv channel protein	C-terminal amino acid 'chain' length	Normalized expression level (IFS/µm^2^) × 10^5^^c^	Mean number of clusters per cell (1/µm^2^) × 10^–2^^d^	Mean fraction of channel-associated signal within clusters (× 10^–3^)^e^	Mean cluster signal intensity (~ channel density) (IFSc/µm^2^) × 10^5^^f^	Channel-PSD-95 binding energy (kcal/mol)^g^
*Shaker B* ∆135	9	60.5 ± 0.15	1.3 ± 0.02	0.15 ± 0.03	3.7 ± 0.02	− 11.68 ± 0.10
*Shaker B* ∆120	24	58.4 ± 0.29	2.7 ± 0.03	1.89 ± 0.77	8.3 ± 0.03	− 11.48 ± 0.28
*Shaker B* C_L_ replaced by I_S_^a^	39	53.6 ± 0.31	8.0 ± 0.06	5.21 ± 1.63	10.7 ± 0.01	− 11.28 ± 0.26
*Shaker B* ∆94	50	49.3 ± 0.15	20.9 ± 0.13	12.07 ± 1.12	18.7 ± 0.01	ND
Shaker B ∆83	61	76.6 ± 0.97	64.2 ± 0.33	95.80 ± 10.60	44.8 ± 0.01	− 10.62 ± 0.17
*Shaker B* C_L_ replaced by I_L_^a^	88	68.0 ± 0.84	47.8 ± 0.23	61.80 ± 5.81	36.4 ± 0.01	− 10.50 ± 0.29
*Shaker B* ∆40	104	77.5 ± 0.98	35.6 ± 0.16	22.99 ± 1.30	26.8 ± 0.01	− 10.02 ± 0.21
WT *Shaker A*	106	56.4 ± 0.15	38.4 ± 0.24	26.27 ± 2.18	26.3 ± 0.01	− 10.12 ± 0.07
WT *Shaker B*	144	54.2 ± 0.36	19.9 ± 0.19	12.29 ± 3.46	15.5 ± 0.01	− 9.76 ± 0.24
*Shaker B* ^R^C_L_^b^	144	46.9 ± 0.31	17.4 ± 0.10	9.25 ± 0.79	14.8 ± 0.01	− 9.65 ± 0.34

In all columns, values and standard errors are reported.

^a^Chimeric Kv *Shaker B* channel variants where the short (S) or long (L) versions of the intrinsically disordered inactivation (I) 'chain' replace the original long C-terminal clustering (C) 'chain'. See ref.^20^.

^b^A Kv channel variant where the amino acid sequence of the long C-terminal clustering 'chain' was (C_L_) reversed (^R^C_L_). See ref.^20^.

^c^Normalized to cell area and calculated by integrating the fluorescence signal (IFS) of a given cell and dividing by cell area (see “Methods” section).

^d^Normalized to cell area and obtained by dividing the cluster number in a given cell by cell area.

^e^Calculated by dividing the integrated IFS within clusters (IFSc) of a given cell by the total cell IFS (see “Methods” section).

^f^Evaluated by dividing the IFSc of a given cluster by cluster area.

^g^Evaluated by surface plasmon resonance binding analysis of the indicated Kv channel 'chain' length with the PDZ domains of PSD-95. See ref.^20^.

## Discussion

Ion channels are not randomly distributed in membranes but rather are targeted to unique membrane domains, where they are organized into complexes along with auxiliary subunits, interacting signaling and anchoring proteins, and sometimes even with other ion channel proteins^33–35^. This multi-protein complex context affects the functional properties of the channel, as compared to the isolated channel context. The formation of channel clusters, however, reflects yet another dimension in the regulation of channel function. Cluster formation corresponds to an active mechanism responsible for bringing many ion channel molecules together, probably to within interaction distances. Evidence is constantly accumulating, showing that such spatial proximity affects the conduction properties of the channel^36–39^ and that changes in ion channel cluster density may lead to changes in action potential properties, such as frequency and/or firing pattern^7–9^. Given the emerging importance of ion channel density for electrical signaling, understanding the mechanism underlying cellular ion channel clustering and its regulation are of prime value. When such mechanistic knowledge is available, interesting insight is possible, as demonstrated here for the case of the *Shaker* Kv channel protein.

**Figure 6:**
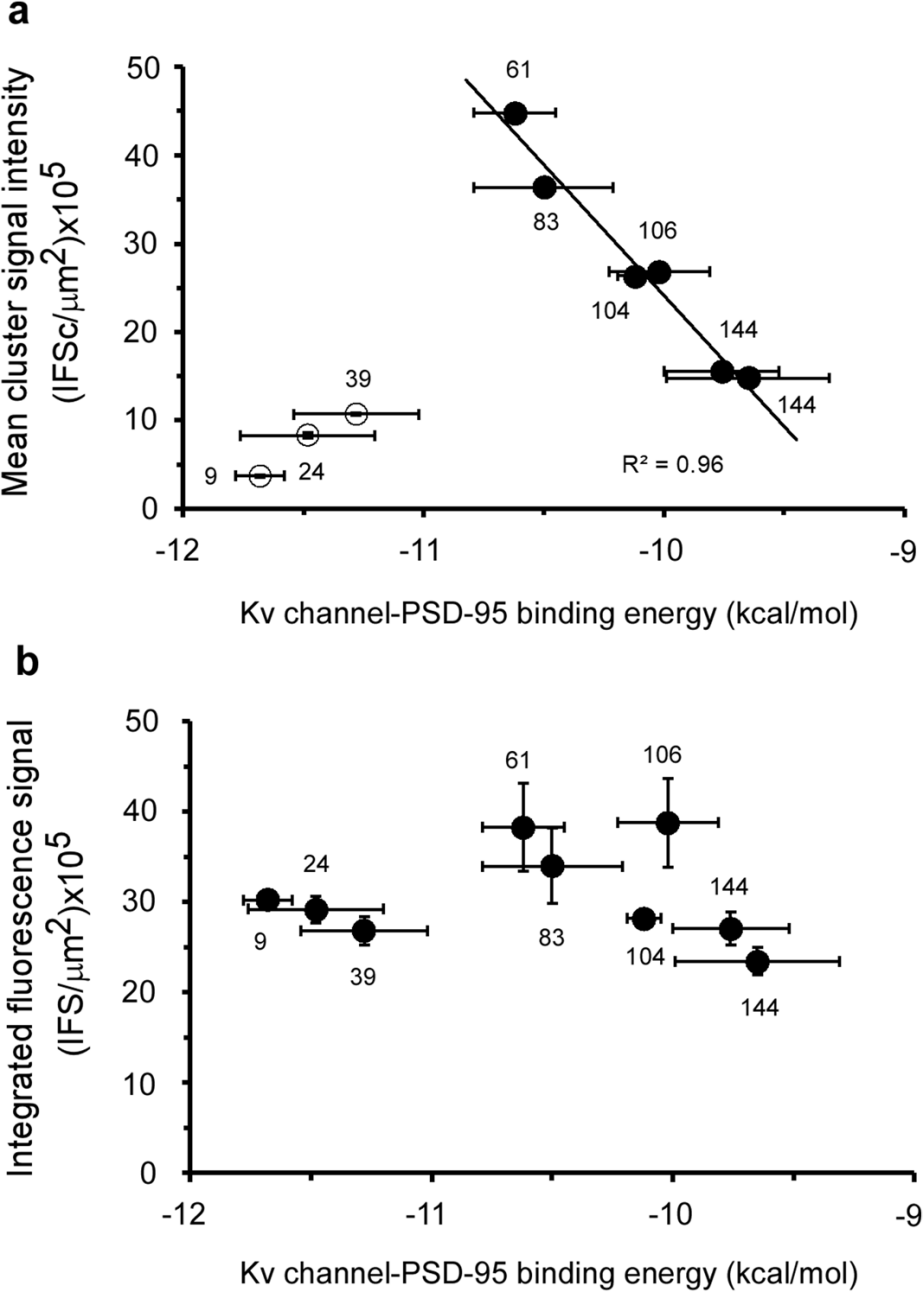
Entropy-based regulation of cluster Kv channel density. (**a**) Correlation plot relating the mean cluster ion channel density and PSD-95 binding affinity of the different Kv channel ‘chain’-length variants. The solid curve represents a linear regression between the compared quantities (*R*^2^ = 0.96) observed for Kv channel variants presenting chain lengths of 61 amino acids or higher. (**b**) Dependence of the mean channel expression levels of the different Kv channel variants on Kv channel-PSD-95 affinity (expressed as binding energy). The number next to each data point corresponds to C-terminal ‘chain’ length.

In the current report, we performed high-resolution confocal microscopy cell imaging combined with clustering analysis to test whether Kv channel C-terminal channel ‘chain’ length, shown to affect thermodynamic aspects of PSD-95 binding, also determines cellular aspects of PSD-95-mediated channel clustering, in particular, membrane channel expression levels and cluster ion channel density. We found that while cell expression levels of the *Shaker* Kv channel does not depend on ‘chain’ length, the number of clusters per cell, the fraction of channel proteins targeted to clusters and the density of ion channels within clusters, all exhibit a bell-shaped dependence on ‘chain’ length. The inverse linear relation between the fraction of channels in clusters and ‘chain’ length (up to 61 amino acids) is in line with previous findings based on other heterologous cell expression systems^17^, and correlates with the role of PSD-95 in stabilizing the channel within the membrane by suppressing endocytosis-mediated channel internalization^40^. The chaperoning role of PSD-95^41^, manifested in the increase of total channel expression^14^, might reflect direct and stoichiometric (1:1) interaction with the Kv channel protein during channel transport to the plasma membrane and appears to be independent of 'chain' length (Fig. 3a, Supplementary Fig. S5c). A different scenario occurs, however, in the context of channel clustering, where several Kv channel molecules may bind to a single PSD-95 protein. In this case, shortening of the Kv channel ‘chain’ monotonically increases the magnitude of all cellular attributes of channel clustering up to a certain ‘chain’ length, from which point further shortening results in the opposite trend (Fig. 5c, Supplementary Fig. S5a–c). Considering the stoichiometry of the channel-scaffold protein interaction, involving the four ion channel tails and three different PDZ domain of PSD-95, and plausible suggested models for channel clustering^42^, we suggest that this behavior is a manifestation of steric hindrance due to the inability to bring several channel molecules into close proximity on the same of PSD-95 molecule when ‘chain’ length decreases too much. Our results thus suggest that the bell-shaped dependence of channel clustering attributes on ‘chain’ length reflects a tradeoff between thermodynamic considerations controlling the PSD-95-channel interaction and steric hindrance considerations. Support for this assertion is obtained by plotting the mean cluster ion channel density of all the ‘chain’ length variants considered here as a function of the affinity of the same variants to PSD-95 (Fig. 6a). A linear correlation is observed between both quantities up to the maximum point of the bell-shaped mean channel density-'chain' length curve. Higher affinity of the Kv channel to PSD-95 is correlated with denser ion channel clusters. The correlation breaks down, however, for the very short ‘chains’, where steric hindrance effects come into play. No such correlation occurs when PSD-95-mediated total channel cell surface expression is considered (Fig. 6b). Interestingly, the bell-shaped behavior of channel density on ‘chain’ length is reminiscent of the effect of N-terminal ‘chain’ shortening on Kv channel fast inactivation. There, shortening of the ‘chain’ resulted in increases in the rate of channel entry into the fast inactivated state, with too short ‘chains’ decelerating inactivation simply because its becomes harder for the inactivation ‘ball’ motif to reach its receptor site on the open channel pore^18,21^.

Taken together, our results reveal the cellular correlates of the molecular ‘ball and chain’ mechanism for channel-PSD-95 binding, with respect to channel clustering. *Shaker* Kv channel ‘chain’ length determines the density of ion channels within clusters, among other clustering attributes. This scenario further emphasizes the existence of entropy-based regulation of Kv channel clustering that mirrors the thermodynamic entropy signature of the preceding Kv channel-PSD-95 molecular binding reaction^17,20^. Super-resolution single molecule localization microscopy is a natural extension of the present study to test the validity of this assertion as it directly counts channel molecules within such clusters^28,30,35^. Such analysis is currently underway.

What is the relevance of the results obtained here for the prototypical fly *Shaker* Kv channel to mammalian channel clustering? Mammalian Kv channels do not undergo alternative splicing at their C-terminal tails. However, different Kv channel paralogues do exhibit differences in C-terminal chain lengths. Furthermore, phylogenetic inference analyses of the entire Kv channel family revealed that for Kv 1, 3 and 4 channel families but not the Kv 2 family, the property of intrinsic disorder at the C-terminal tail co-evolved with the appearance of the terminal PDZ sequence motif^43^. These results suggest a similar effect of the C-terminal tails of these Kv channels in PSD-95-mediated channel clustering. However, this inference remains to be experimentally validated. Interestingly, a truncation mutation in the human Kv 1.1 channel that deleted the entire C-terminal tail of the channel resulted in the onset of episodic ataxia type 1^44^. Using electrophysiological whole-cell recordings, it was demonstrated that this mutation resulted in a dramatic reduction of potassium current expression, a phenotypic behavior that is in accordance with the probable abolishment of interactions with scaffold proteins, potentially hampering channel clustering^44^.

The proposed ‘ball and chain’ mechanism for *Shaker* Kv channel clustering sheds new light on the roles of the short ‘chain’ *A* and long ‘chain’ *B* alternatively spliced *K*v channel variants in electrical signaling. These variants present high and low affinity to PSD-95, respectively, giving rise to distinct cluster ion channel densities. Thus, spatial and temporal expression of the alternatively spliced short or long C-terminal ‘chain’ versions^45,46^ and/or channel variability resulting from hetero-oligomeric *A* and *B* subunit assembly^47^ would give rise to channels with distinct affinities to PSD-95, in turn, leading to distinct PSD-95-mediated ion channel densities. Such modulation, as demonstrated here, would lead to changes in ion current density at the post-synaptic sites of homo- or hetero-oligomeric channel localization, for example, at the post-synaptic density. This could subsequently lead to changes in action potential transmission, frequency, synaptic growth and plasticity^7–9^. On a final note, the Kv channel model protein discussed here, with its two native spliced variants and mutants thereof, provides a clear example whereby molecular distinctions reflected in the differential interactions of the ‘chain’ variants with PSD-95 translate into functional differences in the context of cellular channel clustering. Understanding the linkage between the two levels of organization and its physiological implications is only possible when knowledge of the molecular mechanism underlying channel-scaffold interaction is available.

## Methods

### Molecular biology and cell culture, transfection and immunostaining

For protein clustering analysis, both the PSD-95 and *Shaker* Kv channel proteins used in the current study are *Drosophila* proteins, with the S97 PSD-95 homologue used. SH-SY5Y neuroblastoma cells (kindly donated by Dr. Debbie Toiber, Ben-Gurion University) were cultured in high-glucose Dulbecco-modified Eagle medium (DMEM) supplemented with 10% fetal bovine serum, 1% penicillin–streptomycin (NyStatin) and 1% l-glutamine (Biological industries) at 37 °C with 5% CO_2_. Cells were transfected in situ on coverslips at 70% confluence with 0.5 µg of either the pcDNA-FLAG Channel or pEGFP-PSD95 expression plasmids in a twelve-well plate using Lipofectamine 2000 transfection reagent (Thermo Fisher Scientific), according to the manufacturer’s instructions. Approximately 20 h after transfection, the cells were washed with PBS and fixed with 4% paraformaldehyde in PBS for 15 min at room temperature. The cells were permeabilized, blocked with 0.5% Triton X-100 and 5% bovine serum albumin in PBS for 1 h and washed with PBS. Immunostaining was achieved by incubating the fixed cells with PBS containing mouse monoclonal anti-FLAG primary antibodies (F1804-Sigma) at a 1:1,000 dilution for 1 h, followed by a 45 min incubation with 1:1,000 diluted anti-mouse Alexa-Fluor 568 secondary antibodies (17543 Abcam) in PBS. The cells were washed and mounted using Fluoromount-G (Southern-biotech).

### Sub-diffraction high-resolution laser-scanning confocal microscopy

To assess cellular Kv channel expression and clustering, the basal membrane area of fixed SH-SY5Y neuroblastoma cells transfected to express either native or artificial ‘chain’-length channel variants, with or without the PSD-95-GFP scaffold protein, was imaged using a Zeiss LSM880 inverted laser-scanning confocal microscope (Jena, Germany) equipped with an Airyscan high-resolution detection unit^48^ and under identical acquisition conditions. A Plain-Apochromat 63x/1.4 Oil DIC M27 objective was used, and parameters were set to avoid pixel intensity saturation and to ensure Nyquist sampling in the XY plane (~ 150 nm resolution). In our measurements, the actual axial resolution of the microscope was ~ 350 nm^29^. Detection of FLAG-tagged Kv channel and PSD-95-GFP fusion protein was achieved by focusing directly on the cover slip-attached basal membrane plane and measuring the respective red and green fluorescence signals using a 561 nm DPSS laser with a BP 570–620 emission filter and a 488 nm argon laser with a BP 495–550 emission filter, respectively. Although focusing on the basal membrane plane, given the axial resolution of the microscope, the observed signals report on what is found immediately near or on the plasma membrane. Four lines of evidence support this assertion. First, the Kv channel protein is an integral membrane protein. Second, the Kv channel has been previously imaged in other expression systems and was found to reside in or immediately near the membrane, presenting either a diffuse or speckled pattern, depending on the absence or presence of the PSD-95 scaffold protein, respectively, as was also observed here (see text)^12,14,17^. Third, either the red channel-associated or the green PSD-95-associated signal decreased significantly when descending in the Z direction and towards the intracellular milieu (not shown). Fourth, when imaging wild type *Shaker A* or *B* samples in a total internal reflection (TIRF) mode using the super-resolution microscope (the Zeiss Elyra inverted wide-field fluorescence microscope), the red Kv channel-associated signal appeared to be completely in the TIRF zone (100 nm) (Supporting Information Fig. S1). Taken together, all signals reported here reflect near- or within-membrane channel clustering and intra-cellular aggregation due to artifacts stemming from over-expression (see text for further discussion).

### Kv channel cellular expression and clustering analyses

Quantitative assessment of channel expression and clustering of either native or artificial ‘chain’ length Kv channel variants was performed by evaluating the following four parameters: The normalized expression level, the fraction of fluorescence signal targeted to clusters, the number of channel clusters per cell and cluster fluorescence signal intensity. Importantly, this latter parameter directly reflects ion channel density within clusters. For calculating the first parameter, the red, channel-associated fluorescence signal along each imaged cell area (*n* = 30) was integrated and the total fluorescence signal (IFS) value (reflecting channels residing in or between clusters) was divided by the cell area to yield the normalized PSD-95-mediated cell expression level for a particular channel variant. The other three PSD-95-mediated clustering attributes of the different Kv channel variants were quantitatively assessed using a two-step clustering metrics methodology. Briefly, Kv channel clustering sites within each co-transfected cell image (detected using the red, channel-associated signal), were identified and counted using the 'Spot Counter' plug-in (https://github.com/nicost/spotCounter/) of the ImageJ analysis program^49^. This plug-in assists in detecting local fluorescence maxima by scanning the image with a box of pre-defined size. Local maxima are accepted when the maximum is higher than a user-defined number over the average of the four corners of the box. Later, images in which cluster “spots” were identified and assessed by the 'Threshold-based segmentation' ImageJ macro-code (provided in Supplementary Text S1) that automatically identifies clustering sites, defines their borders and calculates cluster area and integrated florescence intensity within each cluster (IFSc, corrected for the image background signal). For each of the ten Kv channel ‘chain’ variants, 30 cells were analyzed and a total of around 350–7,500 clusters were measured to determine cluster area size and the integrated fluorescent signal. Cluster ion channel density is then evaluated by fluorescent signal intensity obtained by dividing both quantities. The normalized cluster number per cell was calculated by dividing the number of channel clusters within a cell by cell area. The fraction of channels in clustering sites was calculated by integrating the channel-associated red fluorescence signal of all clusters (IFSc) and then dividing by the total red fluorescence signal of the same cell (IFS). For all cell expression and clustering parameters evaluated here, mean values and standard errors were obtained and are reported in Table 1. Since all experiments reported here were performed using identical conditions, comparison of the clustering parameters for different Kv channel 'chain' length variants provides important insight into the mechanism of Kv channel density regulation.

### Statistical analysis

Differences in the mean values for cell expression levels, the fraction of clustered channels, the number of cluster per cells and channel cluster density among the different Kv channel ‘chain’ length variants were assessed using an ANOVA test and assuming normal distributions. The mean cluster ion channel density of the different variants was also compared assuming non-normal distributions of the cluster channel densities (Supplementary Fig. S3). For this purpose, a generalized linear model analysis (GLM) was performed with γ distribution assumed for the cluster ion channel density variable and a log link function between the linear predictor and the mean of the distribution function. The Wald Chi square test was used to examine the null hypothesis that the mean cluster area sizes of the different channel variants are the same. Rejection of the null hypothesis was based on *p* values smaller than 0.001. As in the simple ANOVA test used, here again, the mean value of cluster ion channel density for all the different channel variants were found to be different (*n* = 350–7,000; *p* < 0.00001 in an ANOVA test, except for the control channel pair exhibiting identical ‘chain’ lengths (*p* = 0.1 or higher).

## Supplementary information

Supplementary information

## Data Availability

All data generated or analyzed during this study are included in this published article (and its Supplementary Information files).
