# Supplementary material for: Molecular and cellular correlates in Kv channel clustering: entropy-based regulation of cluster ion channel density
Source: Sci Rep. 2020 Jul 9;10:11304. doi: 10.1038/s41598-020-68003-4 (PMC7347538; doi:10.1038/s41598-020-68003-4)
Supplement: Supplementary file 1 — Supplementary information [file 41598_2020_68003_MOESM1_ESM.pdf]

Molecular and cellular correlates in Kv channel clustering: Entropy-based regulation of cluster ion channel density

Limor Lewin[1], Esraa Nsasra[1], Ella Golbary[1], Uzi Hadad[2], Irit Orr[1] and Ofer Yifrach[1,*]

[1]Department of Life Sciences and the Zlotowski Center for Neurosciences, Ben-Gurion University of the Negev, P.O.B. 653, Beer Sheva 84105, Israel

[2]Ilse Katz Institute for Nanoscale Science & Technology, Ben-Gurion University of the Negev, P.O.B. 653, Beer Sheva 84105, Israel

*

Supplementary Information

The Supplementary information reported here includes Supplementary Figures S1-S5 and Supplementary Text S1.

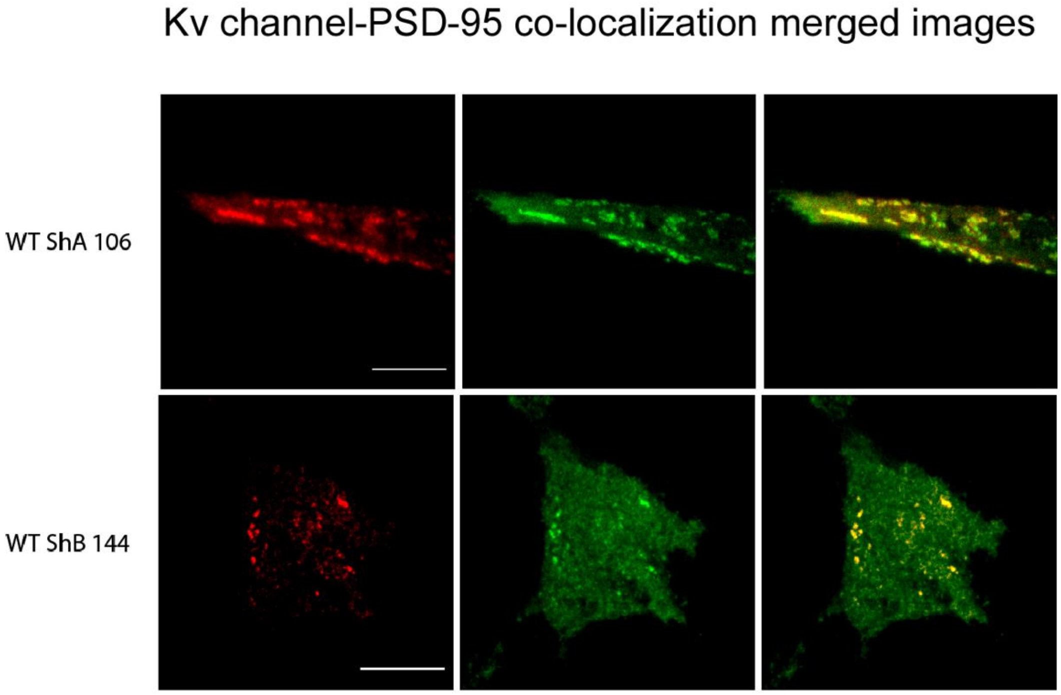

Supplementary Figure S1. Cell expression and clustering patterns of the native *A* and *B Shaker* Kv channel variants. Super-resolution TIRF mode confocal microscopy images of SH-SY5Y neuroblastoma cells co-expressing the native *Shaker A* or *B* Kv channel variants with PSD-95-GFP. For each cell, three images are shown, with the red channel-associated and green PSD-95-associated fluorescent signals presented in the left and middle columns, respectively. The merged image for each cell is shown in the right column.

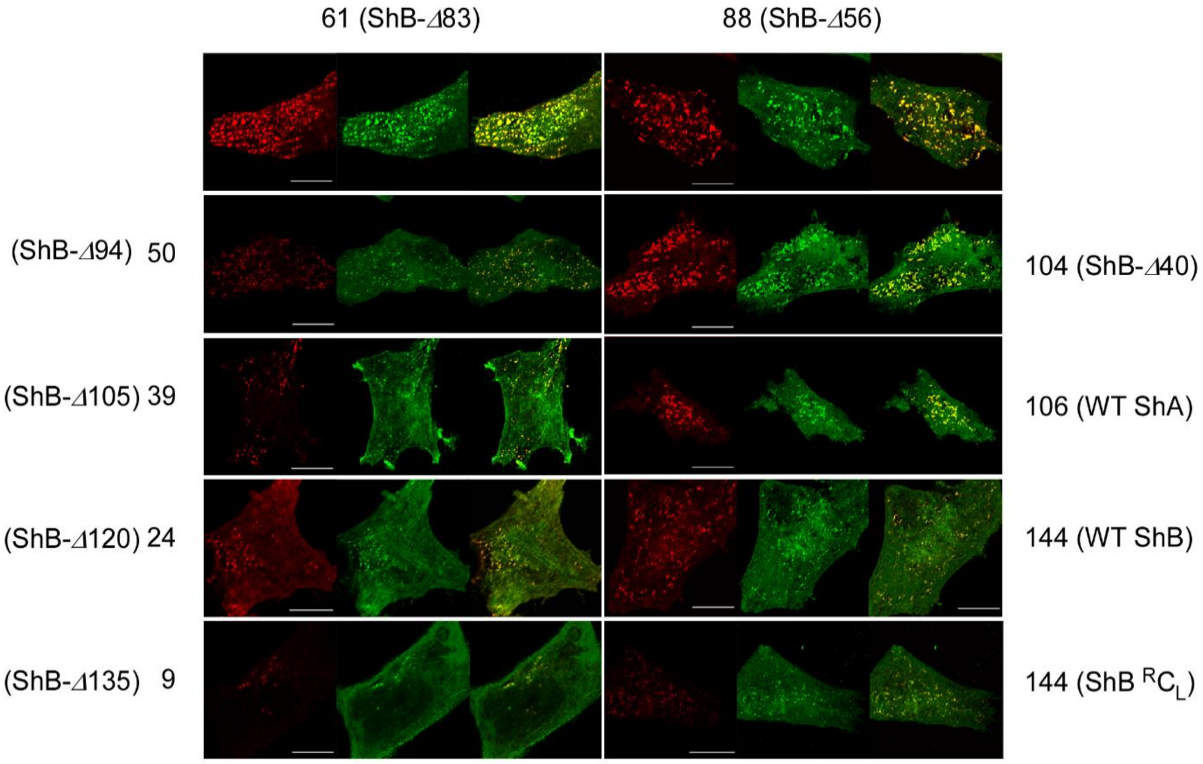

Supplementary Figure S2. Cell expression and clustering patterns of the *Shaker* Kv channel 'chain' length variants. Typical high-resolution confocal microscopy images of cells co-expressing PSD-95-GFP and either the native or mutant channel variants. For each cell, three images are shown, with the red channel-associated and green PSD-95-associated fluorescence signals presented in the left and middle columns, respectively. The merged image for each cell is shown in the right column. Scale bars correspond to 10 μm. Numbers next to each channel notation indicate C-terminal amino acid 'chain' length.

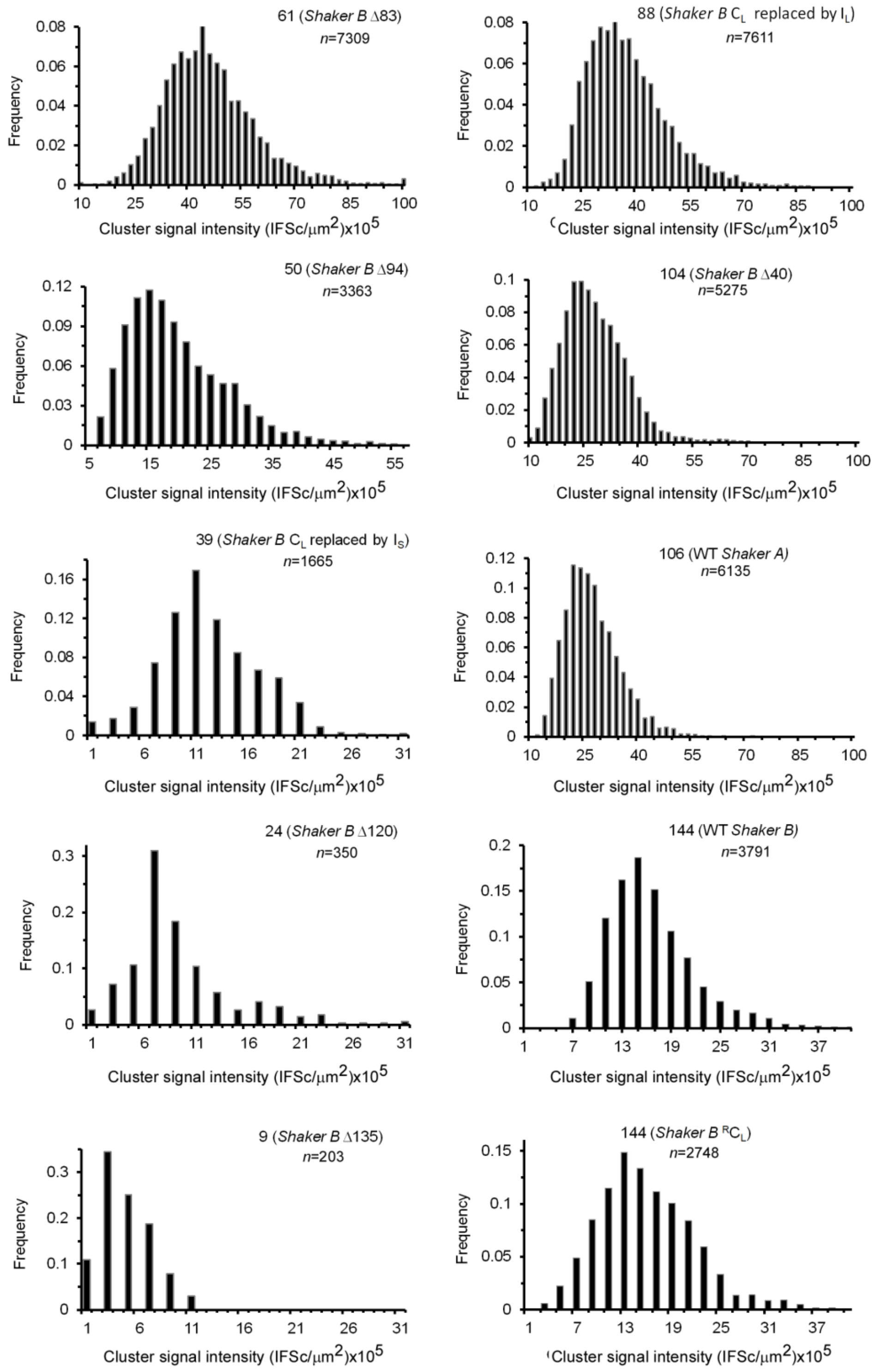

SI Figure 3

Supplementary Figure S3. Cluster Kv channel density distribution of the different ‘chain’-length variants, as evaluated by cluster signal intensity. Distributions in steps of 2 (x10^5) IFS/μm^2 are presented (*n*=200-7,600 clusters, as indicated). The number next to each channel variant indicates ‘chain’ length.

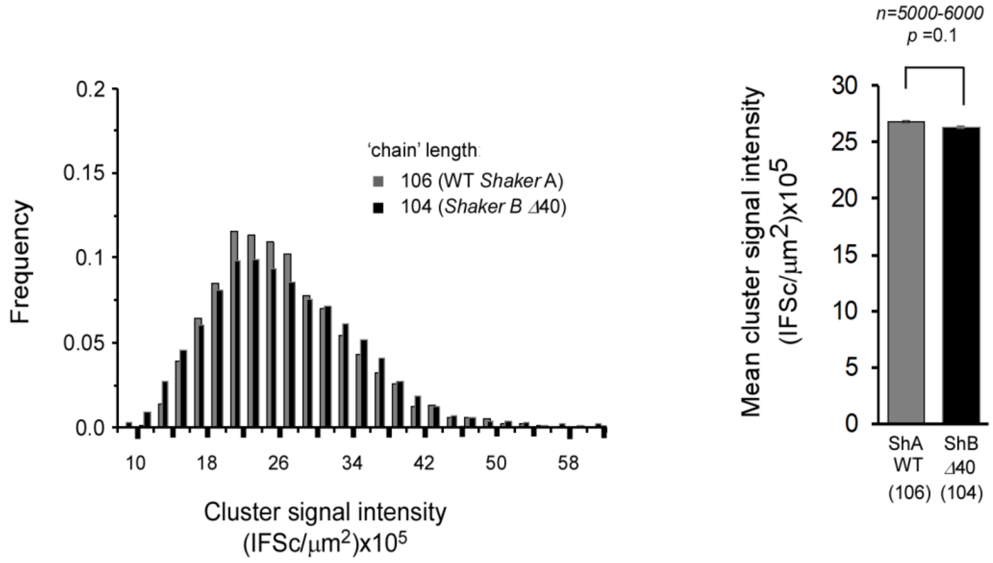

SI Figure 4

Supplementary Figure S4. Comparison of cluster signal intensity (reflecting ion channel density) of the *Shaker* channel variants exhibiting very similar C-terminal chain lengths and composition. Comparison of cluster Kv channel density distribution of the wild type *Shaker A* and mutant *Shaker B ⊿40* deletion channels presenting 106 and 104 amino acid-long 'chains', respectively. The mean values of cluster ion channel density for the two variants are compared in the panel to the right.

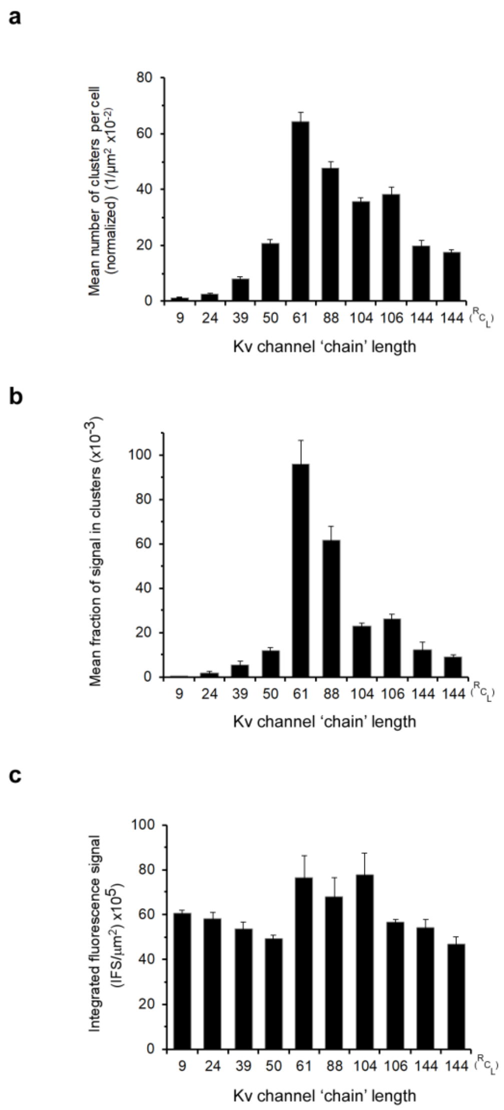

SI Figure 5

Supplementary Figure S5. Comparison of cell expression and clustering attributes of the different Kv channel 'chain' length variants. (a) Comparison of the mean number of clusters per cell (normalized to cell area) among the different Kv channel 'chain' length variants. (b) Comparison of the mean fraction of channels targeted to clustering sites among the different Kv channel 'chain' length variants. (c) Comparison of mean cell expression levels (normalized to cell membrane area) of the different Kv channel 'chain' lengths. Differences in the parameters compared in panels a and b were all found to be statistically significant, other than for variants of identical or similar chain length (ANOVA test; n=30; $p \leq 0.05$). No statistically significant differences were observed among variants with respect to surface Kv channel expression levels.

Supplementary Text S1: The imageJ macrocode used for off-line clustering analysis, as described in the Methods and in the main text

```
// imagej-macro "spotMeasures" (Herbie G., 12. Dec. 2018) requires( "1.52i" )
;run("Close All");
 //close all open imagesinput = File.openDialog("Open Image");
 //prompt to select image file to analyse dirOutput = getDirectory("Choose Save Folder");
// prompt user to select folder to save tables setBatchMode(true);
//prevent images from shown on monitor run("Bio-Formats Importer", "open=[" + input + "]
autoscale color_mode=Default rois_import=[ROI manager] split_channels view=Hyperstack
stack_order=XYCZT"); //open image from input and split channels wait(1000);
 // allow time for image to open 1 sec fileName = File.name;
 //define parameter "fileName" of the current slected image fileNameNoExt =
File.nameWithoutExtension;//print(fileNameNoExt);
//print to Log window the name of the image selectWindow(""+fileName+" - C=0");
//select the RED channel of the image. assume that RED is
Ch0setOption("BlackBackground", true);run("Set Measurements...", "area mean standard
integrated median limit redirect=None decimal=6");run("Clear Results");run("Subtract...",
"value=9998");
//subtract because of Airyscan backgroundrun("Duplicate...",
"title=temp");selectWindow("temp");run("Gaussian Blur...",
"sigma=10");imageCalculator("Subtract create", ""+fileName+" -
C=0","temp");selectWindow("Result of "+fileName+" - C=0");setAutoThreshold("Otsu
dark");run("Convert to Mask");run("Watershed");run("Analyze Particles...",
"add");close("temp");close("Result of "+fileName+" - C=0");selectWindow(""+fileName+" -
C=0");
//select the RED channel of the image. assume that RED is Ch0roiManager("Show
All");roiManager("multi-measure measure_all append");run("Summarize");run("From ROI
Manager");run("Labels...", "color=white font=12 draw");roiManager("Show
All");setBatchMode(false);run("To ROI Manager");selectWindow("Results");
//select the table of results from "Measure" saveAs("Measurements", dirOutput +
fileNameNoExt +"Results.csv");
 //save results to file selectWindow("Results"); run("Close"); exit();
// modified from imagej-macro "spotMeasures" (Herbie G., 12. Dec. 2018)
```
